# Renal outcomes and predictive value of dyslipidemia in patients with IgA nephropathy: a retrospective cohort study

**DOI:** 10.3389/fmed.2026.1695909

**Published:** 2026-04-15

**Authors:** Yiping Ruan, Qiaoyun Huang, Fuyuan Hong, Miao Lin, Chen Wang, Fayang Lian, Fang Cao, Guokai Yang, Lanting Huang, Xuejing Chen

**Affiliations:** 1 Department of Nephrology, Fujian Provincial Hospital, Fuzhou University Affiliated Provincial Hospital, Shengli Clinical Medical College of Fujian Medical University, Fuzhou, Fujian, China; 2 Department of Computer Engineering, Fuzhou University Zhicheng College, Fuzhou, Fujian, China; 3 Department of Pathology, Fujian Provincial Hospital, Fuzhou University Affiliated Provincial Hospital, Shengli Clinical Medical College of Fujian Medical University, Fuzhou, Fujian, China; 4 Epidemiology and Clinical Research Unit, Fujian Provincial Hospital, Fuzhou University Affiliated Provincial Hospital, Shengli Clinical Medical College of Fujian Medical University, Fuzhou, Fujian, China

**Keywords:** characteristics, dyslipidemia, IgA nephropathy, outcome, prediction

## Abstract

**Background:**

Dyslipidemia is common in chronic kidney disease (CKD), including immunoglobulin A (IgA) nephropathy (IgAN), and may be associated with renal prognosis; however, the value of dyslipidemia in IgAN remains insufficiently assessed. The objective of our study was to investigate clinicopathological characteristics and renal outcomes in IgAN patients with dyslipidemia and to evaluate the prognostic value of lipid abnormalities.

**Methods:**

This cohort study included 458 primary IgAN patients for a retrospective analysis. The clinicopathological features and renal outcomes were recorded. In univariate and multivariate models, the associations between dyslipidemia and renal outcomes, as well as dyslipidemia-associated pathological features, were analyzed.

**Results:**

Patients with dyslipidemia—defined as a total cholesterol of ≥5.2 mmol/L, triglycerides of ≥1.7 mmol/L, or low-density lipoprotein cholesterol [LDL-C] of ≥3.4 mmol/L—exhibited elevated complement levels and worse clinical characteristics with regard to blood pressure, proteinuria, kidney function, and glomerulosclerosis; tubular atrophy/interstitial fibrosis (T1–2), crescents, and vascular lesions were more common. A multivariate logistic regression revealed that T1–2 and arterial intimal fibrosis were significantly associated with dyslipidemia. After a mean follow-up of 54.7 months, dyslipidemia (*p* = 0.001), especially abnormalities in total cholesterol (*p* = 0.016) and triglycerides (*p* = 0.001), was significantly associated with poorer renal survival, and renal survival was not improved after lipid-lowering therapies. In addition to estimated glomerular filtration rate (eGFR) and arterial intimal fibrosis, dyslipidemia is an independent predictor for renal survival in multivariate Cox analyses (model 1: HR, 2.206; 95% CI, 1.127–4.317; *p* = 0.021; model 2: HR, 2.085; 95% CI, 1.059–4.106; *p* = 0.034).

**Conclusion:**

IgAN patients with dyslipidemia exhibited more severe clinicopathological features. Tubular atrophy/interstitial fibrosis and arteriosclerosis/arteriolosclerosis were closely associated with dyslipidemia. Dyslipidemia not only indicates adverse renal outcomes but also serves as an independent prognostic predictor.

## Background

Immunoglobulin A nephropathy (IgAN), recognized as the leading cause of primary glomerular disease globally, exhibits pathologically dominant IgA deposition in the glomerular mesangium. While regional disparities epidemiologically exist, IgAN accounts for approximately 50% of primary glomerular diseases in China and significantly elevates the risk of end-stage renal disease (ESRD) (1). Remarkably, ERSD occurs in approximately 40% of patients approximately 20 years after diagnosis (2).

Generally, levels of renal function and proteinuria in patients with chronic kidney disease (CKD), including IgAN, can affect the levels and properties of circulating lipids, including triglycerides, low-density lipoprotein cholesterol, and others (3). Dyslipidemia is also a potentially modifiable cardiovascular risk factor in CKD. Accordingly, Kidney Disease: Improving Global Outcomes (KDIGO) released a comprehensive clinical practice guideline for lipid management in CKD. However, the association between dyslipidemia and renal outcomes in CKD, particularly in IgAN, although previously suggested, has not been well established, and clinical studies have failed to demonstrate significant protective effects of lipid-lowering therapies on renal prognosis (4–6). Moreover, the predictive value of dyslipidemia for renal outcomes in CKD remains unknown.

Until recently, the role of dyslipidemia in IgAN has not been adequately evaluated in follow-up trials. In this single-center, retrospective study, we utilized our cohort to investigate clinicopathological characteristics and renal outcomes in IgAN patients with dyslipidemia, as well as the prognostic value of dyslipidemia, in Eastern China.

## Materials and methods

### Patients

A total of 458 patients (age >14 years) with biopsy-confirmed primary IgAN were enrolled at Fujian Provincial Hospital from January 2010 to October 2021. Patients with fewer than eight glomeruli on the biopsy or with secondary causes of mesangial IgA deposition, such as Henoch–Schönlein purpura, systemic lupus erythematosus, and liver disease, were excluded.

### Data collection

Baseline demographic and clinicopathological data collected at biopsy included the following: age, sex, medical history, systolic and diastolic blood pressure, serum creatinine, blood urea nitrogen (BUN), serum albumin, uric acid, serum cholesterol, triglycerides, high-density lipoprotein cholesterol (HDL-C), low-density lipoprotein cholesterol (LDL-C), urinary red blood cell (RBC) count, proteinuria, serum IgA, serum C3, serum C4, and treatment modalities.

Dyslipidemia was defined as total cholesterol of ≥5.2 mmol/L, serum triglycerides of ≥1.7 mmol/L, or LDL-C of ≥3.4 mmol/L. Ever-smokers were defined as individuals who currently smoked or had a previous smoking history of at least one cigarette per day for at least 6 months. Habitual alcohol consumption was reported by the patients and defined as more than one standard drink per week, equivalent to 14 g of alcohol. Hypertension was defined as blood pressure of ≥140/90 mmHg; blood pressure measurements were repeated twice in a patient who was relaxed and seated in a chair for > 5 min, with measurements taken in the patient’s right arm. The mean arterial pressure (MAP) was defined as diastolic pressure plus one-third of the pulse pressure. Proteinuria was measured by a 24-h urine protein collection. Estimated glomerular filtration rate (eGFR) was calculated using the Chronic Kidney Disease Epidemiology Collaboration (CKD-EPI) equation. Lipid-lowering therapy primarily involved statins, with fibrates reserved for hypertriglyceridemia. Treatment modalities for renin–angiotensin system inhibitors (RASi) included angiotensin-converting enzyme inhibitors (ACEIs), angiotensin receptor blockers (ARBs), or both. Patients received optimized supportive therapy with RAS blockade to achieve target blood pressure (BP) (urine albumin excretion of <30 mg/24 h, BP of ≤ 140/90 mmHg; urine albumin excretion of ≥30 mg/24 h, BP of ≤ 130/80 mmHg). Immunosuppressive therapy was defined as receiving treatment with corticosteroids and/or immunosuppressants after biopsy. Corticosteroid therapy referred to oral prednisone initiated at 0.5–1 mg/kg daily and tapered over 6–8 months. Immunosuppressants indicated cyclophosphamide administered at a total dosage of 6 to 8 g over 6–8 months.

Renal biopsy specimens from all patients were routinely divided for immunofluorescence microscopy, light microscopy, and electron microscopy. The paraffin-embedded sections were stained with hematoxylin and eosin, periodic acid–Schiff, silver methenamine, and Masson’s trichrome. All renal biopsy results were reviewed independently by two renal pathologists according to the most recent Oxford classification. Pathological features of the updated Oxford classification were defined as follows: a mesangial score of ≤0.5 (M0) or >0.5 (M1); endocapillary hypercellularity absent (E0) or present (E1); segmental glomerulosclerosis absent (S0) or present (S1); tubular atrophy/interstitial fibrosis ≤25% (T0), 26–50% (T1), or >50% (T2); and cellular/fibrocellular crescents absent (C0), present in at least one glomerulus (C1), or present in >25% of glomeruli (C2). Additional pathological features included global glomerulosclerosis, tuft necrosis, interstitial inflammation, arterial intimal fibrosis, arteriolar hyalinosis, and IgA immunofluorescence in glomeruli.

### Outcome

The time of renal biopsy was defined as the baseline. The primary outcome of interest was a composite kidney outcome defined as (1) end-stage renal disease (ESRD) or (2) a > 50% decline in eGFR. ESRD was defined as eGFR <15 mL/min/1.73 m^2^ or the initiation of dialysis or transplantation.

### Statistical analysis

Continuous variables were expressed as means ± standard deviation and analyzed using t-tests or Mann–Whitney U-tests. Categorical variables were described as frequencies with percentages and analyzed using the χ^2^ test. Renal survival was analyzed using the Kaplan–Meier method and compared using the log-rank test. Associations between pathological features and dyslipidemia were also analyzed using a univariate logistic regression analysis, followed by a multivariate logistic regression analysis. The results were expressed as odds ratios (ORs) with 95% confidence intervals (CIs). Univariate and multivariate Cox regression analyses were used to determine the independent predictive value of dyslipidemia for renal survival. The clinicopathological features were included in the univariate Cox regression analysis, and only the features significantly associated with renal survival were considered in the multivariate regression analysis. The results were expressed as hazard ratios (HRs) with 95% confidence intervals (CIs). A *p*-value of <0.05 was considered statistically significant. To validate the results, the propensity score-matched sample was established from the original cohort. Propensity score matching was performed using the following parameters: nearest-neighbor matching (1:1) with a caliper of 0.02 and covariates of age, sex, smoking, eGFR, MAP, proteinuria, and serum albumin. All statistical analyses were performed using IBM SPSS 27.0 (IBM Corp., Armonk, NY, USA).

## Results

### Clinical characteristics

At the time of biopsy, the average age of 458 patients recruited in our study was 33.2 ± 10.0 years. The mean eGFR was 87.7 ± 33.7 mL/min/1.73 m^2^. According to the Kidney Disease Outcomes Quality Initiative classification, 352 (76.9%) patients had stage 1 or 2 chronic kidney disease, 79 (17.2%) patients had stage 3, and 27 (5.9%) patients had stage 4 chronic kidney disease. The mean follow-up time was 54.7 ± 20.4 months. During follow-up, the majority of patients received RASi. A total of 137 (29.9%) patients received corticosteroids, and 12 patients (2.6%) received immunosuppressants.

Overall, 256 of the 458 (55.9%) patients presented with dyslipidemia. Of these, 181 (39.5%) patients had total cholesterol of ≥5.2 mmol/L, 148 (32.3%) had triglycerides of ≥1.7 mmol/L, and 159 (34.7%) had LDL-C of ≥3.4 mmol/L. At the time of renal biopsy, patients with dyslipidemia had higher levels of cholesterol (*p* < 0.001), triglycerides (*p* < 0.001), and LDL-C (*p* < 0.001), but HDL-C levels did not differ significantly. These patients were older age (*p* < 0.001) and had higher blood pressure levels, including systolic blood pressure (*p* < 0.001), diastolic blood pressure (*p* < 0.001), and MAP (*p* < 0.001); serum creatinine (*p* < 0.001), BUN (*p* < 0.001), uric acid (*p* < 0.001), proteinuria (*p* < 0.001), C3 (*p* < 0.001), and C4 (*p* < 0.001); as well as lower eGFR (*p* < 0.001) and serum albumin (*p* = 0.001). The proportion of patients with hypertension (*p* < 0.001) was also higher in the dyslipidemia group. There were no significant differences in sex, history of tonsillitis, ever-smokers, habitual alcohol consumption, diabetes, hematuria, and serum IgA levels between the two groups. In addition, among the 256 patients with dyslipidemia, those who received lipid-lowering therapy had more severe proteinuria (1.48 ± 1.50 vs. 1.09 ± 1.08 g/day, *p* = 0.026) and lower levels of serum albumin (36.5 ± 6.3 vs. 38.7 ± 6.4 g/L, *p* = 0.017) than those who had not received lipid-lowering therapy. There were no significant differences in other clinicopathological features between patients with lipid-lowering therapy and those without lipid-lowering therapy.

The length of follow-up was similar, but compared with patients without dyslipidemia, patients with dyslipidemia received more corticosteroids (*p* < 0.001) and lipid-lowering therapies (*p* < 0.001) and had similar use of immunosuppressants. The rate of renal endpoints was higher in patients with dyslipidemia (6.4 vs. 16.0%, *p* = 0.002) (Table 1).

**Table 1:** Clinical and pathological characteristics of IgA nephropathy patients with dyslipidemia.

Parameters	No dyslipidemia (*n* = 202)	Dyslipidemia (*n* = 256)	*P*-value^a^
Age (yr), mean±SD	30.2 ± 8.3	35.5 ± 10.7	<0.001
Male, *n* (%)	88 (43.6)	123 (48.0)	0.339
History of tonsillitis, *n* (%)	44 (21.8)	38 (14.8)	0.054
Ever-smokers, *n* (%)	9 (4.5)	15 (5.9)	0.503
Habitual alcohol consumption, *n* (%)	2 (1.0)	1 (0.4)	0.430
Diabetes, *n* (%)	1 (0.5)	2 (0.8)	0.706
Hypertension, *n* (%)	50 (24.8)	113 (44.1)	<0.001
Systolic BP (mmHg), mean±SD	121.0 ± 16.3	130.0 ± 19.0	<0.001
Diastolic BP (mmHg), mean±SD	76.6 ± 11.8	82.2 ± 13.4	<0.001
MAP (mmHg), mean±SD	91.4 ± 12.4	98.1 ± 14.2	<0.001
Uric acid (μmol/L), mean±SD	337.1 ± 117.7	394.4 ± 125.3	<0.001
Serum creatinine (μmol/L), mean±SD	93.1 ± 58.6	110.0 ± 60.5	<0.001
Serum creatinine >133 μmol/L, *n* (%)	25 (12.4)	63 (24.6)	0.001
eGFR (ml/min/1.73 m^2^), mean±SD	96.5 ± 31.8	80.8 ± 33.7	<0.001
BUN (mmol/L), mean±SD	5.91 ± 4.28	6.92 ± 3.37	<0.001
Proteinuria (g/day), mean±SD	0.67 ± 0.89	1.20 ± 1.21	<0.001
Serum albumin (g/L), mean±SD	40.4 ± 4.1	38.1 ± 6.5	0.001
Cholesterol (mmol/L), mean±SD	4.21 ± 0.55	5.94 ± 1.58	<0.001
Triglycerides (mmol/L), mean±SD	0.95 ± 0.33	2.28 ± 2.12	<0.001
LDL-C (mmol/L), mean±SD	2.60 ± 0.47	3.68 ± 1.29	<0.001
HDL-C (mmol/L), mean±SD	1.27 ± 0.31	1.27 ± 0.61	0.097
RBC in urine≥ 2+, n (%)	85(42.1)	96(37.5)	0.320
Serum IgA (g/L), mean±SD	3.02 ± 0.91	2.94 ± 0.93	0.467
Serum C3 (g/L), mean±SD	0.96 ± 0.18	1.08 ± 0.19	<0.001
Serum C4 (g/L), mean±SD	0.22 ± 0.07	0.25 ± 0.08	<0.001
Length of follow-up (months), mean±SD	56.4 ± 21.9	53.3 ± 19.2	0.087
Treatment, *n* (%)			
Lipid-lowering therapy	0(0)	67(26.2)	<0.001
Steroids	37 (18.3)	100 (39.1)	<0.001
Immunosuppressant	5 (2.5)	7 (2.7)	0.863
ESRD or doubling serum creatinine, *n* (%)	13 (6.4)	41 (16.0)	0.002
Global glomerulosclerosis, *n* (%)	135 (66.8)	214 (83.6)	<0.001
Tuft necrosis, *n* (%)	18 (8.9)	27 (10.5)	0.559
Mesangial hypercellularity, M1, *n* (%)	186 (92.1)	238 (93.0)	0.718
Endocapillary hypercellularity, E1, *n* (%)	34 (16.8)	49 (19.1)	0.524
Segmental glomerulosclerosis, S1, *n* (%)	81 (40.1)	136 (53.1)	0.006
Tubular atrophy/interstitial fibrosis, T, *n* (%)			<0.001
T0	163 (80.7)	161 (62.9)	
T1	37 (18.3)	87 (34.0)	
T2	2 (1.0)	8 (3.1)	
Crescents, *n* (%)			0.022
C0	126 (62.4)	129 (50.4)	
C1	72 (35.6)	115 (44.9)	
C2	4 (2.0)	12 (4.7)	
Lymphocyte and monocyte infiltration, *n* (%)			0.050
0–25%	171 (84.7)	198 (77.3)	
>25%	31 (15.3)	58 (22.7)	
Arterial intimal fibrosis, *n* (%)	52 (25.7)	111 (43.4)	<0.001
Arteriolar hyalinosis, *n* (%)	30 (14.9)	63 (24.6)	0.010
IgA glomerulus immunofluorescence, *n* (%)			0.978
+/++	111 (55.0)	141 (55.1)	
+++/++++	91 (45.0)	115 (44.9)	

BP, blood pressure; MAP, mean arterial pressure; BMI, body mass index; HDL-C, high-density lipoprotein cholesterol; LDL-C, low-density lipoprotein cholesterol; eGFR, estimated glomerular filtration rate; BUN, blood urea nitrogen; RBC, red blood cell; RAS, renin–angiotensin system; ESRD, end-stage renal disease.

^a^*p*-value: comparison between two groups. A *p*-value of <0.05 was considered statistically significant.

### Pathological characteristics

In general, pathological features, including global glomerulosclerosis (*p* < 0.001), segmental glomerulosclerosis (S1) (*p* = 0.006), tubular atrophy/interstitial fibrosis (T1/2) (*p* < 0.001), crescents (C1/2) (*p* = 0.022), arterial intimal fibrosis (*p* < 0.001), and arteriolar hyalinosis (*p* = 0.010), were more severe in patients with dyslipidemia. There was no difference in tuft necrosis, mesangial hypercellularity (M1), endocapillary hypercellularity (E1), lymphocyte and monocyte infiltration, and IgA immunofluorescence in glomeruli between the two groups (Table 1).

### Dyslipidemia-associated pathological factors

When analyzing associations between dyslipidemia and various pathological parameters, the univariate logistic regression analysis showed that global glomerulosclerosis, S1, T1–2, C1–2, arterial intimal fibrosis, and arteriolar hyalinosis were positively correlated with dyslipidemia. The multivariate logistic regression analysis showed that T1–2 (OR, 1.634; 95% CI, 1.015–2.632; *p* = 0.043) and arterial intimal fibrosis (OR, 1.609; 95% CI, 1.037–2.497; *p* = 0.034) were significantly associated with dyslipidemia (Table 2).

**Table 2:** Associations between pathological features and dyslipidemia analyzed using logistic regression analyses.

Parameters	Univariate analysis		Multivariate analysis^a^	
	OR (95% CI)	*P*-value	OR (95% CI)	*P*-value
Global glomerulosclerosis	2.529 (1.626–3.934)	<0.001	1.623 (0.993–2.653)	0.053
Tuft necrosis	1.205 (0.644–2.257)	0.560		
Mesangial hypercellularity, M1	1.137 (0.565–2.291)	0.719		
Endocapillary hypercellularity, E1	1.170 (0.722–1.895)	0.524		
Segmental glomerulosclerosis, S1	1.693 (1.165–2.459)	0.006	1.166 (0.774–1.755)	0.463
Tubular atrophy/interstitial fibrosis, T1–2	2.466 (1.602–3.798)	<0.001	1.634 (1.015–2.632)	0.043
Crescents, C1–2	1.632 (1.121–2.376)	0.011	1.393 (0.934–2.077)	0.104
Arterial intimal fibrosis	2.208 (1.479–3.297)	<0.001	1.609 (1.037–2.497)	0.034
Arteriolar hyalinosis	1.872 (1.157–3.027)	0.011	1.322 (0.788–2.218)	0.290

CI, confidence interval. *p* < 0.05 was considered significant.

^a^Multivariate model: all pathological parameters significantly associated with dyslipidemia were included.

### Renal outcome

**Figure 1:**
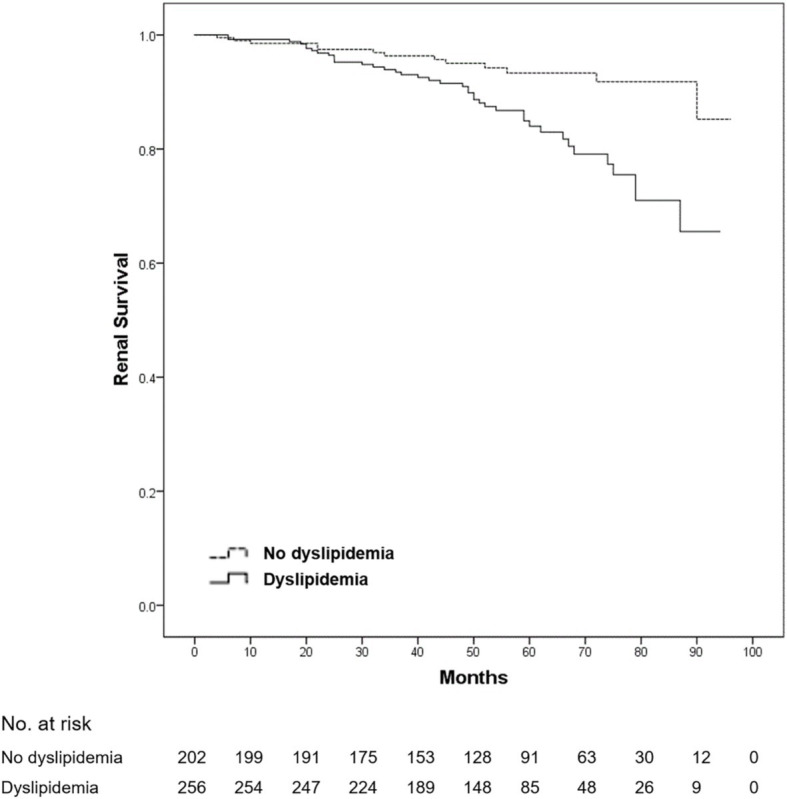
Kaplan–Meier renal survival of IgA nephropathy patients with dyslipidemia. All patients were divided into two groups according to the presence or absence of hyperlipidemia. Compared with the patients without dyslipidemia, the renal survival rate in the patients with dyslipidemia was significantly lower (log-rank test, *p* = 0.001). A *p*-value of <0.05 was considered statistically significant.

**Figure 2:**
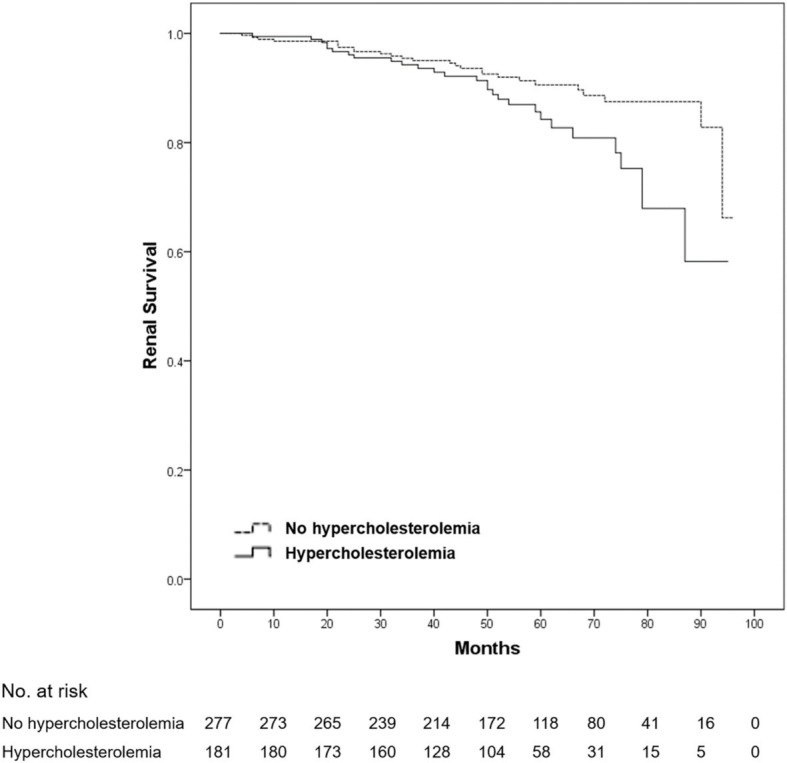
Kaplan–Meier renal survival of IgA nephropathy patients with hypercholesterolemia. All patients were divided into two groups according to the presence or absence of hypercholesterolemia (total cholesterol ≥5.2 mmol/L). Compared with the patients without hypercholesterolemia, the renal survival rate in the patients with hypercholesterolemia was significantly lower (log-rank test, *p* = 0.016). A *p*-value of <0.05 was considered statistically significant.

**Figure 3:**
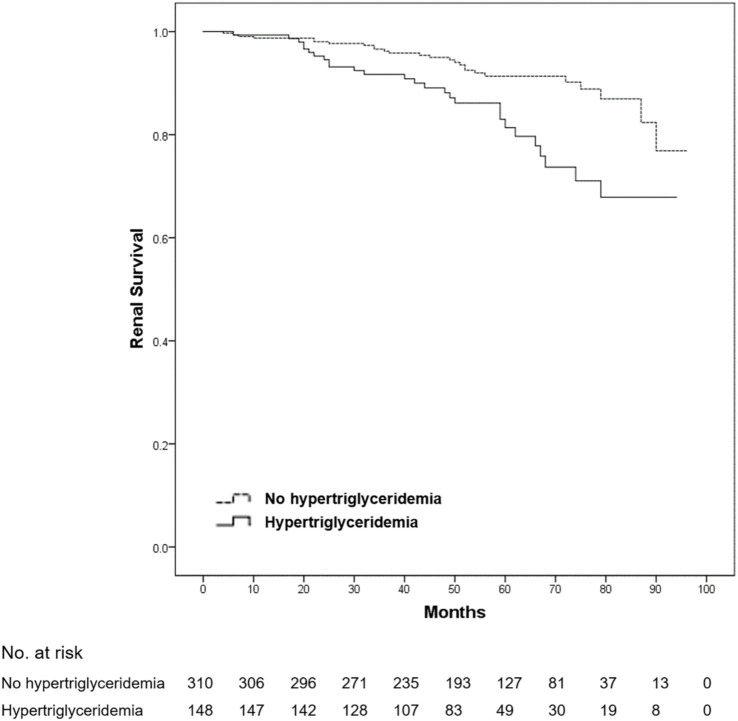
Kaplan–Meier renal survival of IgA nephropathy patients with hypertriglyceridemia. All patients were divided into two groups according to the presence or absence of hypertriglyceridemia (serum triglycerides ≥1.7 mmol/L). Compared with the patients without hypertriglyceridemia, the renal survival rate in the patients with hypertriglyceridemia was significantly lower (log-rank test, *p* = 0.001). A *p*-value of <0.05 was considered statistically significant.

**Figure 4:**
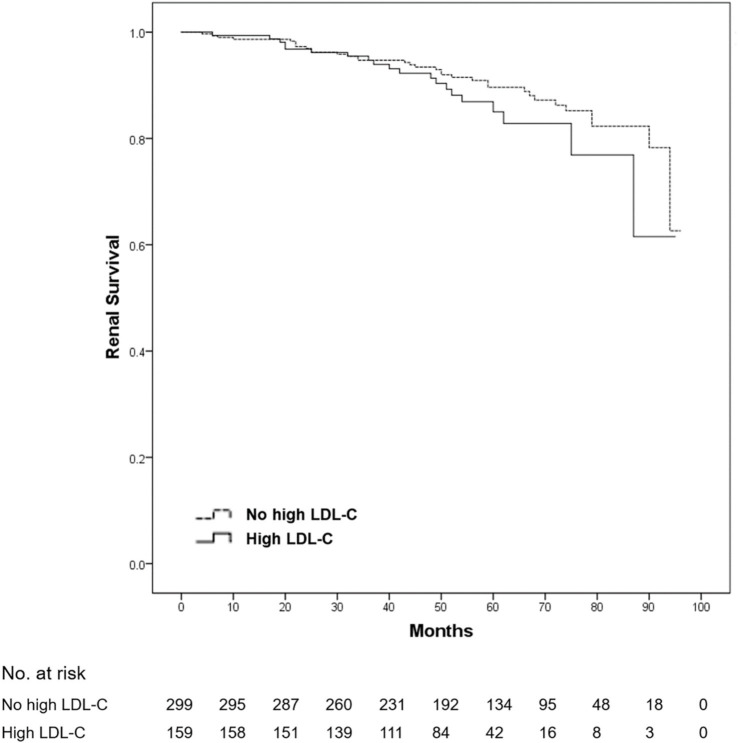
Kaplan–meier renal survival of IgA nephropathy patients with high low-density lipoprotein cholesterol (LDL-C) levels. Patients were divided into two groups based on the presence or absence of elevated LDL-C level (≥3.4 mmol/L). There was no significant difference in renal survival between the two groups (log-rank test, *p* = 0.204). A *p*-value of <0.05 was considered statistically significant.

**Figure 5:**
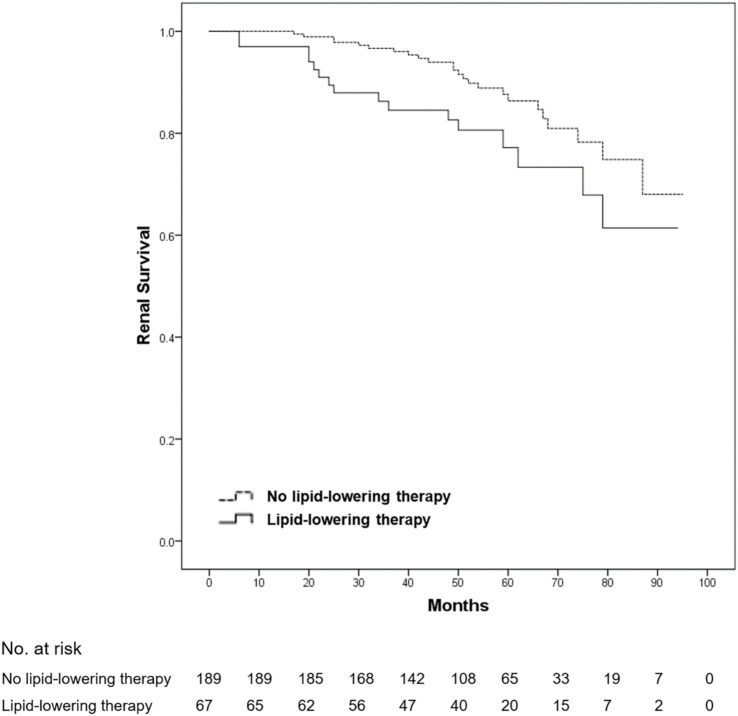
Kaplan–Meier renal survival of IgA nephropathy patients with dyslipidemia according to lipid-lowering therapy. All patients were divided into two groups according to whether they received lipid-lowering therapies. Compared with the patients without lipid-lowering therapies, the renal survival rate in the patients with lipid-lowering therapies was significantly lower (log-rank test, *p* = 0.029). A *p*-value of <0.05 was considered statistically significant.

**Figure 6:**
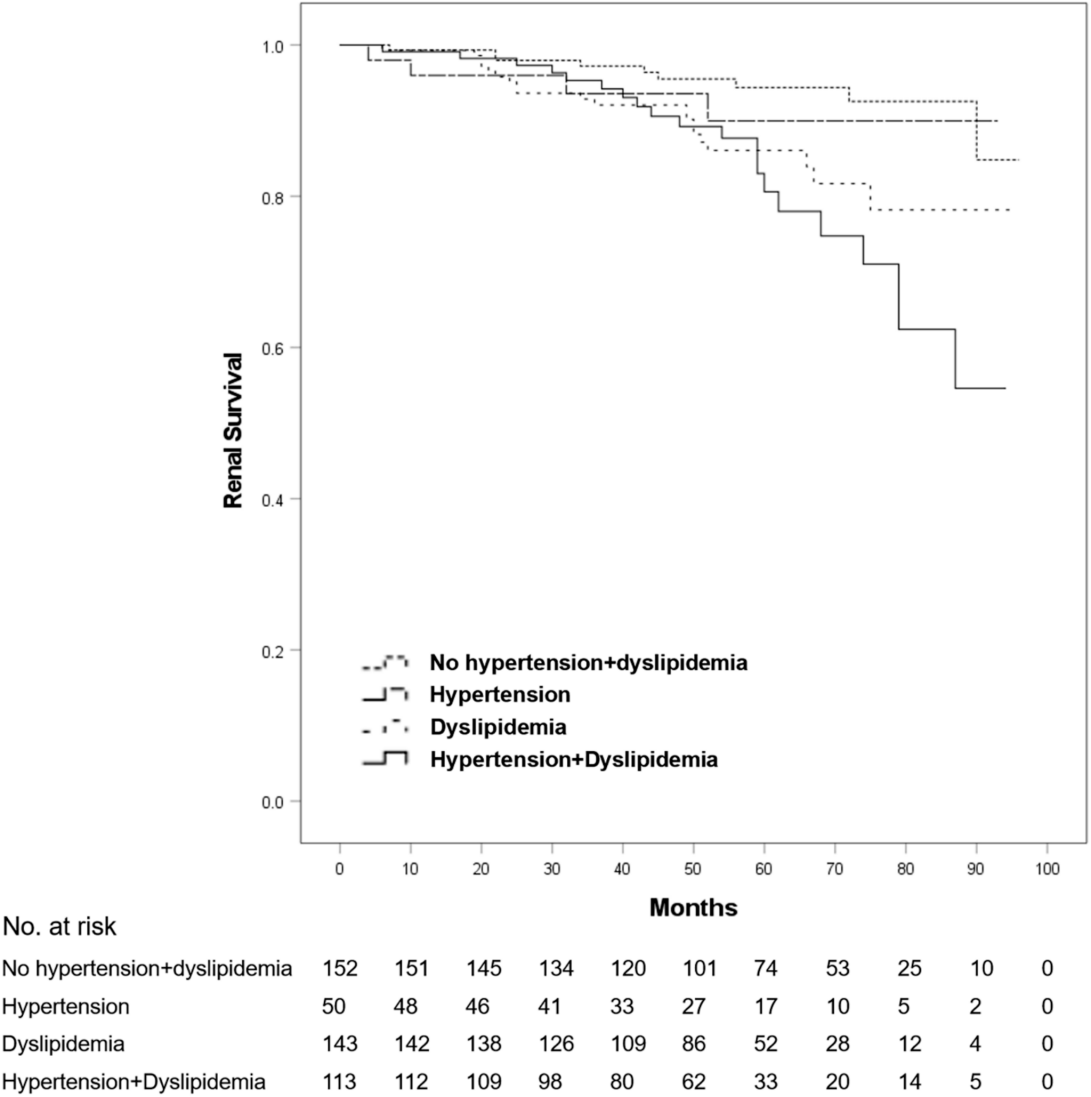
Kaplan–Meier renal survival of IgA nephropathy patients stratified by dyslipidemia and hypertension. All patients were divided into four groups according to whether they presented with dyslipidemia and/or hypertension. Compared with the patients without dyslipidemia and hypertension, the renal survival rates in the patients with dyslipidemia (log-rank test, *p* = 0.011) and with dyslipidemia and hypertension (log-rank test, *p* < 0.001) were significantly lower. No significant differences in renal survival were observed between any other pair of groups. A *p*-value of <0.05 was considered statistically significant.

After a mean follow-up duration of 54.7 months, ESRD or a > 50% decline in eGFR occurred in 54 patients (11.8% of all patients). No death was reported among all patients. Compared with patients without dyslipidemia, the 5-year renal survival rate was significantly lower in those with dyslipidemia (93.3 vs. 84.0%, log-rank *p* = 0.001) (Figure 1). Furthermore, we found that the 5-year renal survival rate was significantly lower in patients with hypercholesterolemia (total cholesterol ≥5.2 mmol/L) (90.6 vs. 84.2%, log-rank *p* = 0.016) (Figure 2) and hypertriglyceridemia (serum triglycerides ≥1.7 mmol/L) (91.4 vs. 81.4%, log-rank *p* = 0.001) (Figure 3). However, there was no significant difference in the renal survival rate between patients with elevated LDL-C levels and those without elevated LDL-C levels (LDL-C ≥ 3.4 mmol/L) during the follow-up (log-rank *p* = 0.204) (Figure 4). In addition, the renal survival rate was similar whether a decreased HDL-C level (HDL-C < 1.0 mmol/L) was present or not (log-rank *p* = 0.138). The renal survival rate was also significantly lower in patients with dyslipidemia (log-rank *p* < 0.001), hypercholesterolemia (log-rank *p* = 0.004), and hypertriglyceridemia (log-rank *p* = 0.022) in the propensity score-matched sample. In patients with dyslipidemia, after lipid-lowering therapies, the 5-year renal survival rate was significantly lower (86.4 vs. 77.2%, log-rank *p* = 0.029) (Figure 5), but in the propensity score-matched sample, the renal survival rates were not significantly different (88.1 vs. 77.2%, log-rank *p* = 0.050). Compared with patients without dyslipidemia and hypertension, the 5-year renal survival rate was significantly lower in patients with both conditions (95.5 vs. 80.6%, log-rank *p* < 0.001) and in those with hyperlipidemia alone (log-rank *p* = 0.011). The 5-year renal survival rates were 90.0% in patients with simple hypertension and 86.0% in patients with hyperlipidemia alone (Figure 6).

### Adjusted predictive value of dyslipidemia

Univariate analyses and multivariate analyses were performed to independently examine the predictive value of dyslipidemia for renal outcomes. The following clinicopathological parameters were used in the univariate analyses: age, sex, MAP, initial eGFR, proteinuria, treatment modalities, pathological features scores based on the most recent Oxford classification, and vascular lesions. In the univariate Cox analyses, dyslipidemia (HR, 2.871; 95% CI, 1.533–5.376; *p* = 0.001), age, ever-smokers, MAP, eGFR, proteinuria >1 g/24 h, endocapillary hypercellularity (E1), tubular atrophy/interstitial fibrosis (T1–2), arterial intimal fibrosis, and arteriolar hyalinosis were strongly associated with renal survival. Nevertheless, after adjusting for clinical parameters in multivariable model 1 and for clinicopathological parameters in multivariable model 2, dyslipidemia (model 1: HR, 2.206; 95% CI, 1.127–4.317; *p* = 0.021; model 2: HR, 2.085; 95% CI, 1.059–4.106; *p* = 0.034), eGFR at baseline (model 1: HR, 0.979; 95% CI, 0.969–0.989; *p* < 0.001; model 2: HR, 0.983; 95% CI, 0.972–0.995; *p* = 0.007), and arterial intimal fibrosis (model 2: HR, 2.180; 95% CI, 1.154–4.120; *p* = 0.016) were independent predictors of renal survival (Table 3).

**Table 3:** Predictive value of dyslipidemia for renal survival assessed using univariate and multivariate Cox regression analyses.

Parameters	Univariable model		Multivariable model 1 ^a^		Multivariable model 2^b^	
	HR (95% CI)	*P*-value	HR (95% CI)	*P*-value	HR (95% CI)	*P*-value
Dyslipidemia	2.871 (1.533–5.376)	0.001	2.206 (1.127–4.317)	0.021	2.085 (1.059–4.106)	0.034
Age (years)	1.027 (1.001–1.054)	0.039	0.995 (0.967–1.024)	0.718	0.989 (0.960–1.019)	0.461
Male	1.597 (0.933–2.733)	0.088				
Ever-smokers	3.158 (1.347–7.400)	0.008	2.164 (0.906–5.173)	0.082	2.603 (1.079–6.279)	0.033
MAP	1.021 (1.004–1.039)	0.016	0.995 (0.975–1.016)	0.662	0.993 (0.973–1.013)	0.490
eGFR (mL/min/1.73 m^2^)	0.978 (0.970–0.987)	<0.001	0.979 (0.969–0.989)	<0.001	0.983 (0.972–0.995)	0.007
Proteinuria (g/day)						
<0.5	Reference					
0.5–1	1.339 (0.680–2.637)	0.399	0.952 (0.478–1.898)	0.890	1.022 (0.509–2.053)	0.951
>1	1.905 (1.014–3.578)	0.045	0.864 (0.430–1.737)	0.682	0.915 (0.455–1.840)	0.803
Steroids therapy	1.352 (0.772–2.366)	0.291				
Immunosuppressant therapy^c^	0.897 (0.124–6.500)	0.914				
M1	0.569 (0.243–1.334)	0.195				
E1	2.119 (1.111–4.044)	0.023			1.551 (0.792–3.039)	0.200
S1	0.750 (0.429–1.310)	0.312				
T1–2	2.502 (1.459–4.291)	<0.001			0.886 (0.434–1.810)	0.741
C1–2	1.336 (0.780–2.288)	0.292				
Arterial intimal fibrosis	3.718 (2.139–6.461)	<0.001			2.180 (1.154–4.120)	0.016
Arteriolar hyalinosis	2.113 (1.108–4.032)	0.023			1.370 (0.689–2.721)	0.369

MAP, mean arterial pressure; eGFR, estimated glomerular filtration rate; M, mesangial hypercellularity; E, endocapillary hypercellularity; S, segmental glomerulosclerosis; T, tubular atrophy/interstitial fibrosis; C, crescents; CI, confidence interval. *p* < 0.05 was considered significant.

^a^Multivariable model 1: all clinical parameters significantly associated with renal outcomes were included.

^b^Multivariable model 2: all clinical and pathological parameters significantly associated with renal outcomes were included.

^c^Immunosuppressant referred to cyclophosphamide.

In addition, the prognostic performance of various lipid profiles combinations was assessed using the univariable model and the same multivariable models for adjustment, including: (1) abnormal levels of total cholesterol (≥5.2 mmol/L) or triglycerides (≥1.7 mmol/L) (univariable model: HR, 2.472; 95% CI, 1.375–4.444; *p* = 0.002; multivariable model 1: HR, 1.888; 95% CI, 1.000–3.563; *p* = 0.050; multivariable model 2: HR, 1.774; 95% CI, 0.934–3.372; *p* = 0.080) and (2) abnormal levels of total cholesterol (≥5.2 mmol/L), triglycerides (≥1.7 mmol/L), LDL-C (≥3.4 mmol/L), or HDL-C (<1.0 mmol/L) (univariable model: HR, 2.708; 95% CI, 1.362–5.383; *p* = 0.004; multivariable model 1: HR, 1.955; 95% CI, 0.947–4.034; *p* = 0.070; multivariable model 2: HR, 2.029; 95% CI, 0.975–4.224; *p* = 0.058).

In the propensity score-matched sample, dyslipidemia (univariable model: HR, 3.270; 95% CI, 1.574–6.792; *p* = 0.001; model 1: HR, 2.494; 95% CI, 1.166–5.335; *p* = 0.019) was also strongly associated with renal survival in both univariate and multivariate Cox analyses. Lipid profiles combinations 1 and 2 exhibited a significant association with renal survival in the univariate analysis (combination 1: HR, 2.630; 95% CI, 1.365–5.067; *p* = 0.004; combination 2: HR, 3.488; 95% CI, 1.482–8.210; *p* = 0.004), whereas no correlation persisted after adjusting for clinical variables in the multivariate model 1 (combination 1: HR, 1.985; 95% CI, 0.996–3.958; *p* = 0.051; combination 2: HR, 1.852; 95% CI, 0.924–3.710; *p* = 0.082).

## Discussion

Until recently, only a few studies have comprehensively investigated detailed clinicopathological characteristics and renal outcomes of dyslipidemia in IgA nephropathy (IgAN) and evaluated the prognostic value of dyslipidemia. In this IgAN cohort, we found that at least 55.9% of patients had different types of dyslipidemia. If measured by identical diagnostic criteria for abnormal lipid profiles, the prevalence of dyslipidemia in our study was comparable to that in recent studies (7, 8). Older age, decreased eGFR and serum albumin, and high levels of blood pressure, proteinuria, uric acid, and serum complements, including C3 and C4, were more severe in patients with dyslipidemia. Dyslipidemia was associated with several chronic pathological lesions, including global and segmental glomerulosclerosis, tubular atrophy/interstitial fibrosis, and arteriosclerosis/arteriolosclerosis (refer to arterial intimal fibrosis and arteriolar hyalinosis). In addition, crescents, which had been observed to be partially irreversible in several studies, were also associated with dyslipidemia. A multivariate logistic regression analysis revealed that vascular and tubulointerstitial lesions were pathological features independently associated with dyslipidemia. IgAN patients with dyslipidemia presented with more severe clinicopathological manifestations, the majority of which were generally consistent with previous reports despite differences in lipid profiles across these studies, including our study (7–9).

Notably, elevated complement levels have been observed in IgAN patients with dyslipidemia (8, 10). For instance, serum complement C3, which was produced primarily by the liver and also found in adipocytes, endothelial cells, and activated macrophages, has been considered to be associated with metabolic disorders such as dyslipidemia (10). Several complement proteins, including C3, C3a-desArg, C4, and C9, bind to lipoprotein particles in plasma and play important roles in their metabolism (11, 12). Complement cascade activation has a well-established pathogenic role in kidney disease, and both the alternative and mannose-binding lectin pathways are implicated in IgAN (13). Furthermore, prior studies, including the present study, have demonstrated associations between intrarenal vascular lesions, arteriolar C4d deposition, and dyslipidemia (14, 15). More research on the association between dyslipidemia and complement activation is required. On the other hand, although dyslipidemia is recognized to be involved in direct nephrotoxicity, inflammatory response, and complement activation (16, 17), taking into account the lack of improvement in renal outcomes with statin-based lipid-lowering therapies in prior studies (4, 18), it remains incompletely understood whether dyslipidemia aggravates the renal pathological alterations in IgAN. Even so, it should be recognized that, regardless of whether lipid-lowering therapies are administered, the poor renal outcomes in patients with dyslipidemia are likely to be largely attributable to other major risk factors, such as more severe proteinuria in our study.

Through univariable and multivariable Cox analyses and validation with the propensity score-matched sample, this lipid profile combination, defined as total cholesterol of ≥5.2 mmol/L, serum triglycerides of ≥1.7 mmol/L, or LDL-C of ≥3.4 mmol/L, demonstrated the optimal predictive value for renal prognosis. Despite heterogeneity in classification criteria for dyslipidemia across studies, accumulating evidence suggests the association between diverse patterns of lipid abnormalities and renal outcomes in CKD, whereas large-scale studies in IgAN remain absent. The relevance of triglyceride and cholesterol abnormalities to renal outcomes has been indicated in CKD (5, 19) and IgAN (7, 9, 20) in previous studies and ours. The value of LDL-C for renal prognosis has been supported in the CKD cohort (21) but remains controversial in IgAN. A study from China has shown that elevated LDL-C level was a predictive factor for the prognosis of IgAN (22), which contradicts our study. A U-shaped association has been observed between serum HDL-C levels and adverse renal outcomes in a large cohort of CKD from the Republic of Korea (23). However, to date, the prognostic value of HDL-C in IgAN, which was not confirmed in our cohort, has not been reported by analogous investigations. In children with IgAN, dyslipidemia has also been considered a risk factor for progression in a retrospective cohort study (24). Moreover, it should be noted that our study revealed differential independent predictive values of lipid profile combinations for renal prognosis. In our IgAN cohort, multivariable-adjusted models demonstrated that the combination of elevated triglycerides, cholesterol, and LDL-C showed the better independent predictive capacity for adverse renal outcomes. Unfortunately, our study found that lipid-lowering therapies for patients with dyslipidemia did not improve renal prognosis, which supports findings from prior studies (4). These findings not only facilitate the application of dyslipidemia for the prediction of renal prognosis but also imply that dyslipidemia might be involved in complex immune-related mechanisms that were not yet fully elucidated in IgAN.

Several limitations of this study should be recognized. First, given that IgAN, as well as dyslipidemia, are long-term chronic diseases with racial and geographical variations, the findings of our study may not be applicable to other ethnic groups or countries. Second, as regards the mild clinicopathological features at onset and the influence of treatment on renal prognosis in patients with dyslipidemia, the follow-up time might be insufficient; therefore, a significant difference in renal survival might have been missed, such as a possible difference between patients with elevated LDL-C and patients without elevated LDL-C. No cardiovascular events were observed either. Third, the limited sample size and clinicopathological features with limited follow-up may have introduced some bias in our single-center retrospective cohort study. For instance, after RASi and immunosuppressive therapy, the association between proteinuria, hypertension, and renal outcomes in the analysis could be attenuated. The limited sample size hampered the assessment of optimal cutoff values for each lipid parameter in predicting renal prognosis and the value of lipid-lowering therapies and immunosuppressants. Due to the very small sample size of alcohol consumption and diabetes, the lack of data on dyslipidemia-associated risk factors, including obesity and family history, and details of lipid-lowering therapy regimens, the conclusion on the association between renal prognosis and dyslipidemia, as well as lipid-lowering therapies, might be partially influenced. Taking into account more clinicopathological parameters, further well-designed multicenter cohort studies with longer regular follow-up and larger sample sizes are still necessary to confirm these disputed results.

## Conclusion

IgAN patients with dyslipidemia exhibited more severe clinicopathological features. Tubular atrophy/interstitial fibrosis and arteriosclerosis/arteriolosclerosis were closely associated with dyslipidemia. Dyslipidemia was not only indicative of adverse renal outcomes but also an independent prognostic predictor for renal survival.

## Data Availability

The original contributions presented in the study are included in the article/supplementary material, further inquiries can be directed to the corresponding author/s.
